# Chlorine Concentration Modelling and Supervision in Water Distribution Systems

**DOI:** 10.3390/s22155578

**Published:** 2022-07-26

**Authors:** Ramon Pérez, Albert Martínez-Torrents, Manuel Martínez, Sergi Grau, Laura Vinardell, Ricard Tomàs, Xavier Martínez-Lladó, Irene Jubany

**Affiliations:** 1 Research Center for Supervision, Safety and Automatic Control, Universitat Politècnica de Catalunya, Rambla Sant Nebridi 10, 0822 Terrassa, Spain; ramon.perez@upc.edu; 2 Sustainability Area, Eurecat, Centre Tecnològic de Catalunya, Plaça de la Ciència, 2, 08243 Manresa, Spain; albert.martinez@eurecat.org (A.M.-T.); manuel.martinez@eurecat.org (M.M.); laura.vinardell@eurecat.org (L.V.); xavier.martinez@eurecat.org (X.M.-L.); 3 Department of Mechanical Engineering, Universitat Rovira i Virgili, Av. Països Catalans 26, 43007 Tarragona, Spain; 4 Aigües de Manresa S.A., Plana de l’Om 6, 3r-3a, 08240 Manresa, Spain; sgrau@aiguesmanresa.cat (S.G.); rtomas@aiguesmanresa.cat (R.T.); 5 Department of Mining, Industrial and ICT Engineering, Universitat Politècnica de Catalunya, Av. de les Bases de Manresa, 61-73, 08242 Manresa, Spain

**Keywords:** chlorine, water distribution networks, modelling, supervision, decay model

## Abstract

The quality of the drinking water distributed through the networks has become the main concern of most operators. This work focuses on one of the most important variables of the drinking water distribution networks (WDN) that use disinfection, chlorine. This powerful disinfectant must be dosed carefully in order to reduce disinfection byproducts (DBPs). The literature demonstrates researchers’ interest in modelling chlorine decay and using several different approaches. Nevertheless, the full-scale application of these models is far from being a reality in the supervision of water distribution networks. This paper combines the use of validated chlorine prediction models with an intensive study of a large amount of data and its influence on the model’s parameters. These parameters are estimated and validated using data coming from the Supervisory Control and Data Acquisition (SCADA) software, a full-scale water distribution system, and using off-line analytics. The result is a powerful methodology for calibrating a chlorine decay model on-line which coherently evolves over time along with the significant variables that influence it.

## 1. Introduction

Disinfection is one of the most important steps in water treatment, as it must ensure the microbiological safety of the water generated, not only after treatment, but also throughout the transport process to the consumption point. Many countries use chlorine-based chemicals (sodium hypochlorite, chlorine dioxide, chloramines, etc.) to achieve this objective, as they guarantee the degree of residual disinfection potential that is required by their laws [1]. If required, booster disinfection stations are installed at different points in the network. Their need and best location can be optimized using models and tools based on estimates of the chlorine concentration. Chlorine concentration is precisely one of the most relevant parameters to consider for the water distribution network (WDN) quality management. Although chlorine ensures the absence of pathogens, it is the main cause of the formation of disinfection byproducts (DBPs) [2]. Most of these compounds are toxic or carcinogenic for human health and need to be controlled to ensure drinking water safety [3]. Thus, European legislation limits the concentration of some DBPs in drinking water [4].

Nowadays, given the lack of reliable and applicable models for predicting chlorine behavior, disinfection management is not optimal in most WDNs, since it is based on a point-specific control as opposed to the consideration of the whole network [5]. There are often dark points where the chlorine level may be too low, along with over-chlorination at other points (particularly in summer), with a subsequent increase in both operating costs and DBP concentrations.

The absence of robust models for predicting chlorine behavior in WDNs is fundamentally due to two aspects: (1) the complexity of modelling the hydraulics of the WDNs and (2) the need for on-line quality data. Although authors report good results in chlorine prediction in full-scale networks in some studies [6], the predictions become less accurate when the environmental conditions or the water composition change from those of the calibration. Such a situation is very common in WDNs fed with treated surface water.

Regarding the first aspect, WDNs are highly meshed and complex systems, the behavior of which is difficult to predict. The introduction of flow, level and pressure sensors, and automated metering readers (AMR) for consumption has recently increased the model accuracy [7,8]. Thus, the intense use of a large amount of hydraulic data together with hydraulic models and numerical simulators allow prediction of residence time, which is one of the main parameters needed for successful water quality prediction.

Regarding the second aspect, it is mandatory to obtain information on water quality in the effluent of the drinking water treatment plant and the relevant points in the WDNs in order to predict the behavior of chlorine. Several studies [6,9] base their decay models on parameters that are easily measured on-line, such as temperature, pH, redox potential, conductivity, turbidity, and chlorine concentration. Nevertheless, the calibration and maintenance of these models for their on-line use is seldom performed.

Common models for chlorine modelling in WDNs are first-order linear differential equations [10] such as:

(1) $$\frac{dC}{dt}=K_{b}\cdot C^{\alpha }$$

where *K_b_* is a constant that contains the different parameters and physicochemical phenomena that may affect chlorine decay, such as natural organic matter, inorganic compounds, or temperature [6]. More sophisticated models have also been studied, including second-order models [11].

Furthermore, knowing the effect of the parameters influencing chlorine decay is also important, as this will, in turn, allow the prediction of such decay and, in some cases, the application of corrective measures to reduce its effect.

Equations of different complexity have been used to model the effect of some of these parameters. One of the most influencing and studied parameters is temperature, which is usually based on Arrhenius’s model [12] or other power models. Liu et al. [13] took pH and temperature into account in their models, thus differentiating the effect of HOCl and OCl species on pH. Similarly, Arevalo, in his doctoral dissertation [14], used a model that considered temperature and UV254 as an indication of organic matter. In this case, two decay constants were used, one related to chlorine decay on bulk water and one related to chlorine decay on water close to pipe wall.

Hassan et al. [15] studied the specific case of organic matter adsorbed onto goethite, which is the predominant iron oxide in pipe deposits, in order to see how effectively the presence of organic matter increased the decay rate. Their main conclusions were: (i) an increase in temperature causes an increase in the decay constant and therefore in the decay rate, (ii) the pH has not been seen to greatly affect decay, (iii) a higher initial chlorine concentration leads to a lower decay rate, (iv) a higher organic matter concentration, in general dissolved organic matter (DOC), causes an increase in the rate of decay, (v) an increase in the velocity of the water flow through the pipe causes an increase in the rate of decay, and (vi) the concentration of ammonium, nitrites, iron, and manganese seems to affect the rate of decay, causing its increase.

Chlorine decay first-order equations can also be used in software like EPANET, which has sufficient power to simulate and predict the concentration of chlorine in the network. EPANET is a public domain software for WDN modelling developed by the United States Environmental Protection Agency (US EPA). This software can perform transient simulations of hydraulic behavior and water quality in pressurized pipe networks. In order to properly model water quality and its time evolution at consumer points, it is mandatory to have a reliable hydraulic model of the WDN. However, like any simulation software, EPANET depends on the availability and application of continuous data into robust models. The default built-in models have fixed calibration parameters that are not easily extrapolated in most real cases. The approach some authors take to overcome these barriers is to modify and recalibrate the default models included in the EPANET database based on the real system data to be modelled [16].

Another important aspect when modelling chemical reactions in pipes is the different behavior in wall and in bulk water. In the literature, bulk reactions are usually considered first-order and wall reactions are considered zero-order. Values for the bulk reaction coefficient are usually obtained using laboratory measurements [17]. Nevertheless, changing water characteristics in the network requires updating the model. There are a few approaches for the quality model calibration. This calibration requires a validated hydraulic model and water quality data. This is often carried out in a well-monitored part of the network and then generalized to the whole network [18,19].

This paper focuses on the chlorine decay process and variables that affect it, the models for concentration prediction, and their application within WDNs in a specific case-study. First, an on-line calibration procedure, with available data from the transport network, is adapted to the full-scale system and performed over a long period so that the evolution of the decay parameters can be studied. This model is used to predict the chlorine concentration in the distribution network and validated with discrete monitoring data. The dependence of chlorine decay on the relevant variables is also studied. Finally, this dependence is compared with the evolution of the parameters estimated using the on-line calibration method. The aim is to illustrate how the intense use of models and available data can provide a better understanding of the behavior of chlorine in a WDN and, thus, be used to support decision-making to improve water quality.

## 2. Materials and Methods

### 2.1. Case Study Network

**Figure 1:**
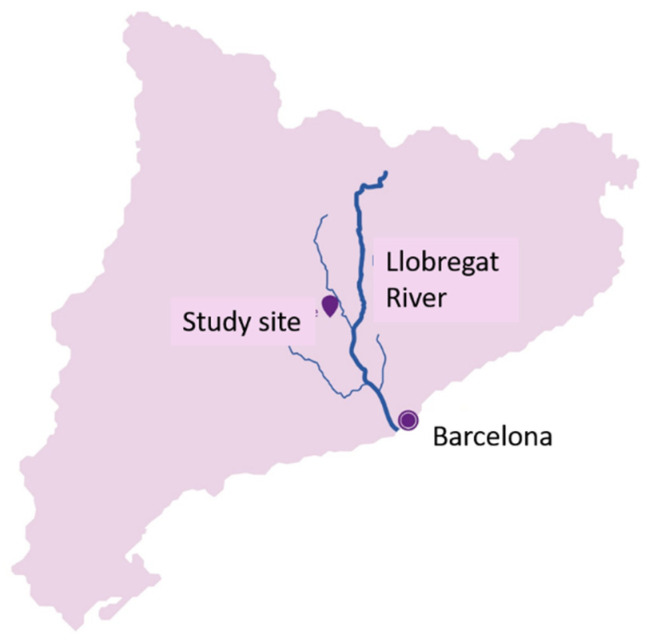
Study site location, a network in Catalonia supplied by the Llobregat River.

The case study in this work is a WDN in Catalonia (Spain) (see location in Figure 1) managed by Aigües de Manresa, who provided the network configuration information and hydraulic and water quality data for a period of 14 months (2017–2018). The water supplied comes from the Llobregat River and goes through a prechlorination step with sodium hypochlorite (Apliclor Water Solutions S.L., Sant Martí Sesgueioles, Spain) or chlorine dioxide generated using sodium chlorite (Apliclor Water Solutions S.L., Sant Martí Sesgueioles, Spain) and hydrochloric acid (Apliclor Water Solutions S.L., Sant Martí Sesgueioles, Spain) (depending on the season), a sand filtration process, and a final disinfection step with sodium hypochlorite.

**Figure 2:**
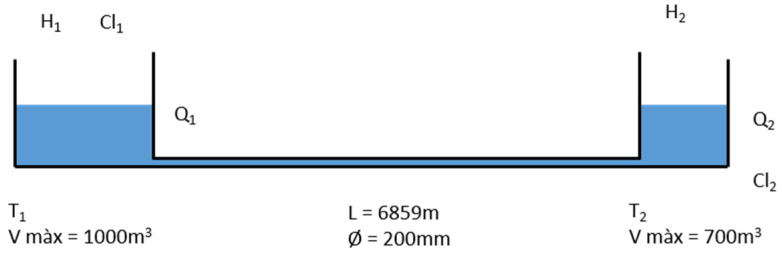
Outline of the network section used for the on-line calibration.

Two parts of the network were used in this study: the transport network and the district metered area (DMA). The transport network (Figure 2) consists of two water storage tanks (T_1_ and T_2_) equipped with sensors for chlorine concentration (input of T_1_ (Cl_1_) and output of T_2_ (Cl_2_)), flowmeters (outflows from the tanks, Q_1_ and Q_2_), and water level (H_1_ and H_2_). Water flows from T_1_ to T_2_ through a 6859 m main. T_2_ is a boosting station with known sodium hypochlorite (Apliclor Water Solutions S.L., Sant Martí Sesgueioles, Spain) addition. The geometry of the tanks (volume) and pipes (length and diameter) are known. Water from T_2_ is distributed to the rest of the network (through Q_2_) of the DMA.

**Figure 3:**
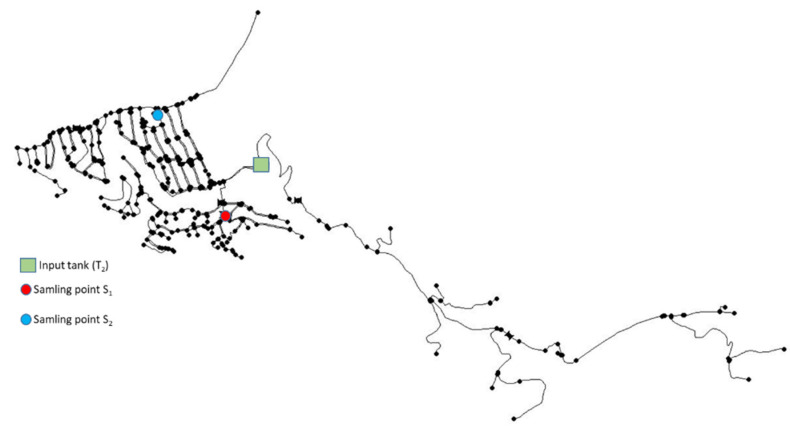
DMA network model in EPANET.

The DMA corresponds to a residential area. The hydraulic model includes 572 nodes and 610 pipes with a total length of 31 km, providing water to 300 consumers. Water flows by gravity. There are two quality-sampling points where the chlorine concentration is measured weekly. Figure 3 presents the model of this DMA visualized in EPANET. The input tank (corresponding to T_2_ in Figure 2) and the two sampling points are highlighted (S_1_ and S_2_).

### 2.2. On-Line Calibration

A very well parametrized system in terms of hydraulics and chlorine concentration (at least at two points) is required to calibrate the chlorine decay constant of a WDN. As in this study, the transport network often fulfils this condition. Therefore, the network used for on-line calibration in this study was the transport network shown in Figure 2. The objective was to find the decay constants for the model that best explained the chlorine concentration measured at the output of T_2_.

The chosen model was a first-order model. Higher-order models could be used with no fundamental changes in the methodology. Equation (2) shows that the chlorine concentration (*Cl_2_*) at the outflow of T_2_ depends on the input chlorine concentration (*Cl_1_*) and the residence time in the system (*t*). The solution of Equation (1) is as follows:

(2) $$Cl_{2}=Cl_{1}\cdot e^{-K_{b}\cdot t}$$

where *K_b_* is the decay constant and *α* is considered as 1.

This dependence is defined by the decay constant *K_b_*, which was calibrated on-line using the measurements available so that it was adapted throughout the year to the different water characteristics and environmental conditions. Estimations were performed on a weekly basis, since some information was only available at this frequency (chlorine dosing in T_2_).

The residence time (RT) in T_1_ was calculated from the hydraulic information available using (3). The weekly mean residence time at tank T_1_ and the pipe was calculated using the flowmeter data (Q_1_) and the volume of this subsystem.

(3) $${\bar{RT}}_{1}=\frac{{\bar{V}}_{1}+V_{pipe}}{{\bar{Q}}_{1}}$$

In order to estimate the mean water volume in T_1_, the level data of the tank (H_1_) and the geometric information was used. The residence time in T_2_ was calculated using the mean values of the volume obtained from the level data (H_2_) and the mean values of the tank effluent (Q_2_), as shown in (4):

(4) $${\bar{RT}}_{2}=\frac{{\bar{V}}_{2}}{{\bar{Q}}_{2}}$$

The chlorine concentration increase due to rechlorination (${\bar{Cl}}_{added}$) was calculated using the added volume of chlorine divided by the mean volume of water treated:

(5) $${\bar{Cl}}_{added}=\frac{\bar{\Delta V_{Cl}}\cdot 143}{\int^{\;}Q_{2}}$$

where ${\bar{\Delta V}}_{Cl}$ is the volume in liters of the concentrated chlorine added weekly to the network and 143 is the concentration of the added chlorine in g/L (value obtained from the conversion of the 15% NaClO to reactive chlorine, see Section S1 in the Supplementary Material).

Finally, a decay *K_b_* constant was calculated which explained the chlorine concentration ${\bar{Cl}}_{2}$ at the outflow of T_2_ given the residence time calculated using (6)

(6) $${\bar{Cl}}_{2}={\bar{Cl}}_{1}\cdot e^{-K_{b}({\bar{RT}}_{1}+R{\bar{RT}}_{2})}+{\bar{Cl}}_{added}\cdot e^{-K_{b}{\bar{RT}}_{T_{2}}}$$

where ${\bar{Cl}}_{2}$ was considered equal to 0.6 ppm, which is the set point of the chlorine control system in the boosting station. The algorithm for *K_b_* calibration is shown in the Supplementary Material (Section S2).

The first order decay model is the most used in the water industry. Its decay constant includes all the dependencies related to environmental and water characteristics. Thus, the continuous updating of this constant is the guarantee of its reliability. The limitation of this methodology is the information required. Hydraulic information, that allows the determination of the residence time, must be available. Multiple chlorine concentration measurements and the exact volume of added chlorine between these measurements are also mandatory data.

### 2.3. Chlorine Decay Calibration and Validation

The chlorine decay first-order model validation for the transport network was carried out using available the on-line data of the chlorine concentration in the output of T2 considering the residence time in this tank. The data used covered the period from February 2017 to April 2018. The chlorine decay model was also validated for its use in the distribution network in the section where chlorine concentration is monitored.

Applying the calibrated decay model directly to the distribution network produced poor results. This was expected, due to the difference between the transport network and the distribution network regarding pipe size, materials, age, etc. To adjust the model, the available data period was divided into two sets: one for training the new distribution quality model and the remaining data for validation. There were 35 samples available in S_1_ and 11 samples in S_2_. Thus, the first 21 samples in S_1_ were used for the training and the remaining ones for the validation. The algorithm used for this calibration is shown in the Supplementary Material (Section S3).

### 2.4. Parametrised Chlorine Decay Model

The decay constant, determined from the available on-line data, evolved clearly throughout 2017. The question arose if this could be due to the effect of the available variables such as temperature, the initial chlorine concentration, the cumulative precipitation, and the turbidity at the drinking water treatment plant or not.

This suggested the idea of analyzing the variables that influence chlorine decay in order to generate an empirical model based on the available independent variables. The availability of considerable data (temporal and spatial) implies dealing with large amounts of data, multiple variables, and experimental noise, which hinders the direct extraction of valuable information.

Principal component analysis (PCA) is a multivariate statistical technique that allows the description of the data according to the variance [20]. This method transforms data in noncorrelated new variables by linear transformation, decreasing the data dimensions. New data description is more condensed and can describe patterns that are hard to identify in multivariable datasets. PCA has already been used to determine the physical and chemical parameters influencing chlorine decay [21]. Therefore, for being a powerful, reliable, and globally accepted tool when dealing with big data, PCA was selected to extract the main trends, patterns, and correlations among the variables (dimensions) [11].

Based on the PCA results, the chlorine decay constant was modelled using the available variables and a potential multiparametric model (7).

(7) $$K_{b}=K\cdot Parameter1^{a}\cdot Parameter2^{b}\cdot Parameter3^{c}\cdot \ldots$$

Specifically, a power model (8) and an Arrhenius model (9) [12] where calibrated using experimental data (temperature in 2017) and *K_b_* obtained from the on-line calibration using the least square error fitting method implemented in the “Solver” function in Excel.

(8) $$K_{b}=K_{power}\cdot \;T^{a}$$

(9) $$K_{b}=A\cdot exp\left(-E_{a}/RT\right)$$

where, *K_power_*, *a*, and *A* are constants, *E_a_* is the activation energy (Jmol^−1^), *R* is the universal gas constant, and *T* is the temperature. Finally, the parametrized chlorine decay model was compared with that obtained in the on-line calibration to assess its coherence throughout the year.

## 3. Results and Discussion

### 3.1. On-Line Calibration

**Figure 4:**
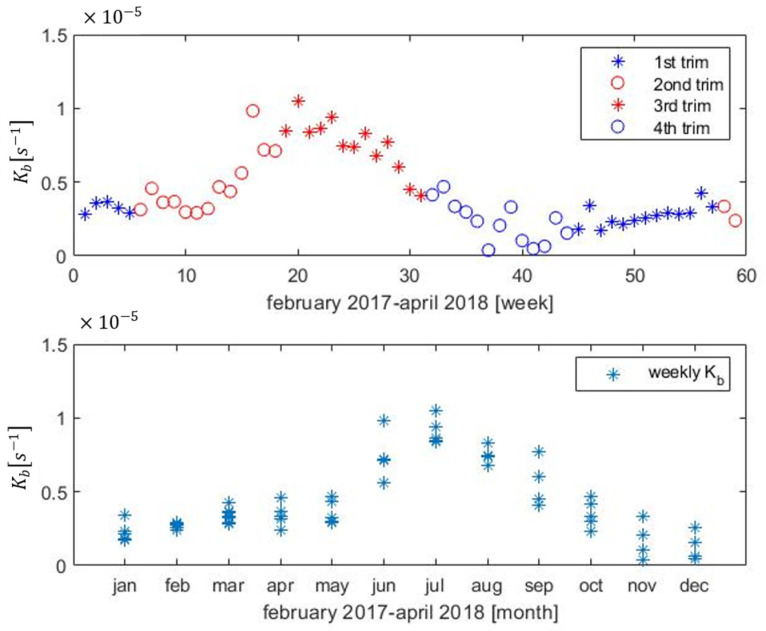
Up: Mean K_b_ calculated weekly indicating the season of the year (by trimester). Down: Data of mean K_b_ grouped by month.

The decay constants *K_b_* obtained are presented in Figure 4. In the upper graphic, the weekly evolution between February 2017 and April 2018, can be observed. A different icon was used for the data of each trimester to clearly identify the season of the year. In the lower graphic obtained, *K_b_* are grouped by month to observe how this parameter evolves throughout the year (some months include estimations of both years). It seems clear that there may be a seasonal variation related to temperature.

### 3.2. Chlorine Decay Validation

**Figure 5:**
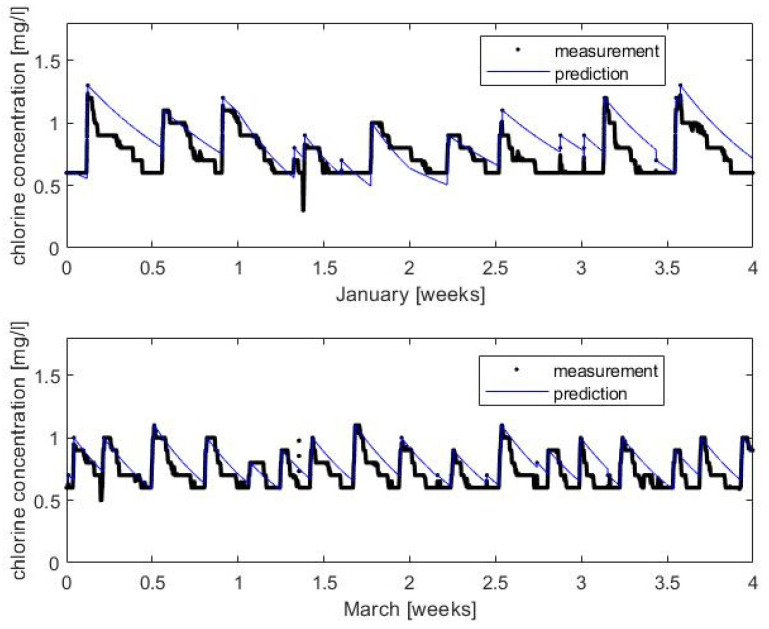
Chlorine decay model validation at the output of T_2_. Up: data from January 2017. Down: data from March 2017. Due to high frequency of sampling measurement, data appears like a thick line.

The calibrated model was applied to the peak episodes observed at the output of T_2_ due to the rechlorination and mixing effect. The dataset used in this validation was not used for the estimation. The dataset for calibration consisted of the mean values corresponding to the stationary state. Figure 5 shows the chlorine concentration data and the model prediction. It can be observed how this high-frequency dynamic is adjusted with the model obtained with the mean values. For this prediction, K_b_ evolves weekly.

**Figure 6:**
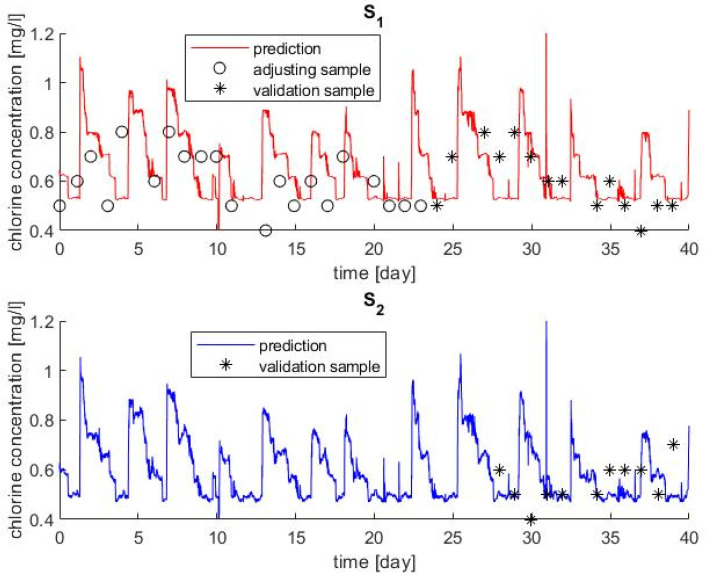
Simulation results for the two sampling points (**S_1_** and **S_2_**) compared with experimental samplings.

For the distribution network simulation, a relation between the transport *K_b_*, estimated by on-line calibration, with the distribution K_b_^*^ was obtained by adjusting the concentration in the training set of chlorine sampling. This relation was applied to the entire period and the predicted concentration was compared with the measurements for the validation set of samples. A total of 40 days were simulated. The decay constant for both the bulk and wall were fitted using the first 21 samples of the chlorine concentration in S1. These are the first samples of upper graphic in Figure 6.

**Figure 7:**
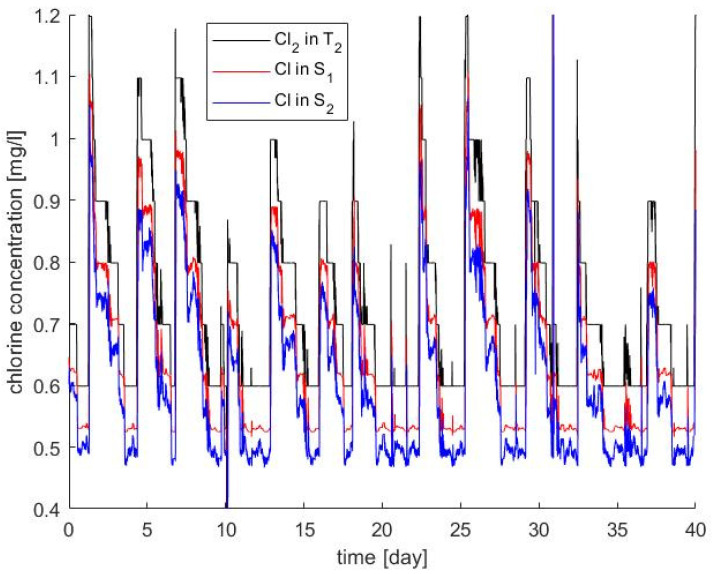
Measured chlorine concentration at the source (T_2_) and prediction at the two sampling points (S_1_ and S_2_).

The result was that both decay constants minimize the error when the original *K_b_* obtained in the transport system was divided by 2, as if the calibrated effect was distributed in the two phenomena (K^*^_b,bulk_ = K^*^_b,wall_ = K_b_/2). The results obtained are compared with the available experimental data in Figure 6. The mean absolute percentage error was 16% for S_1_ (including calibration and validation samples) while it was 17% using only validation samples. Therefore, not significantly different deviations were obtained for the calibration and the validation steps. Graphically, the fit may seem poor; however, the concentration is lower in S_2_ than in S_1_ both in prediction and measurements, and the mean values in both sampling points are coherent between the prediction and measurements. One aspect that may justify part of the mismatching is that the exact hour of the day of the manual measurements was not available and, therefore, each experimental data may not be in its exact position. This difficulty could be overcome with on-line chlorine sensors instead of manual analysis. Figure 7 presents the measured chlorine concentration at the source (T_2_) and the chlorine prediction in the two sampling points (S_1_ and S_2_). The chlorine concentration decreases with the residence time, since the concentration in S_2_ is lower than in S_1_, and both are lower than in T_2_.

**Figure 8:**
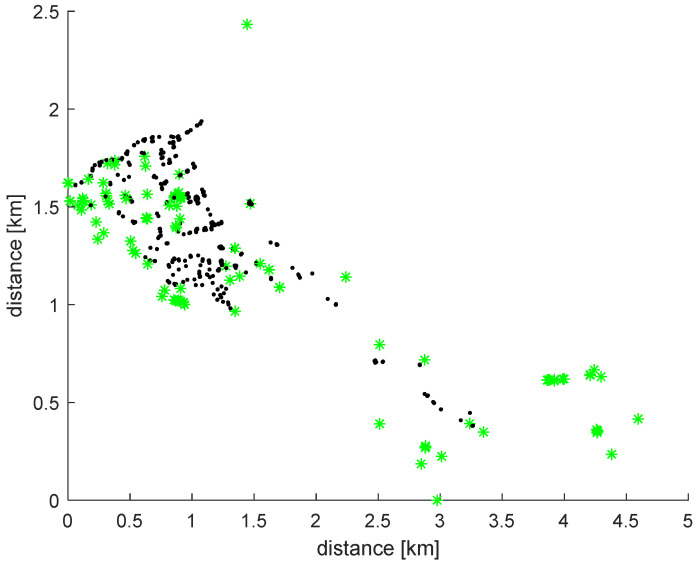
Distribution of network nodes with low concentration (<0.4 ppm, green), acceptable concentration (0.4 ppm > chlorine < 1 ppm, black), and excessive concentration (>1 ppm, red).

Finally, Figure 8 shows the network nodes colored by their chlorine concentration: green, black, or red, depending on whether their concentration is too low, acceptable, or too high, respectively. In fact, no red points exist in this area and period. Such a representation is very useful in order for the network operator to make decisions. Nodes in this figure correspond to those in Figure 3 and are presented with north at the top.

**Figure 9:**
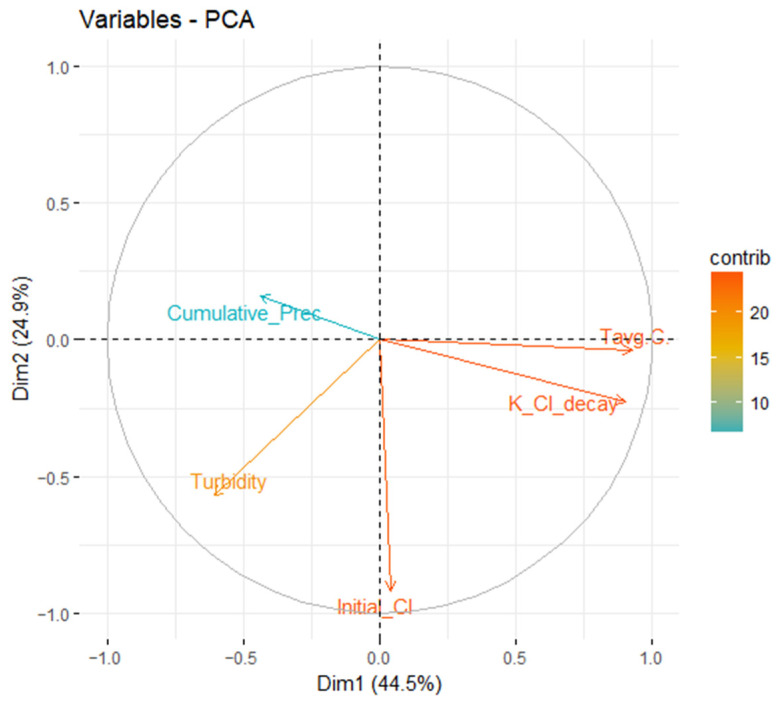
Variables for the two main dimensions of the PCA.

Results from the PCA applied to the decay constant (K_Cl_decay) determined in the distribution network and other data available (temperature as Tavg_C, initial chlorine concentration as Initial_Cl, cumulative precipitation as Cumulative_Prec, and turbidity at the drinking water treatment plant) are shown in Figure 9.

**Figure 10:**
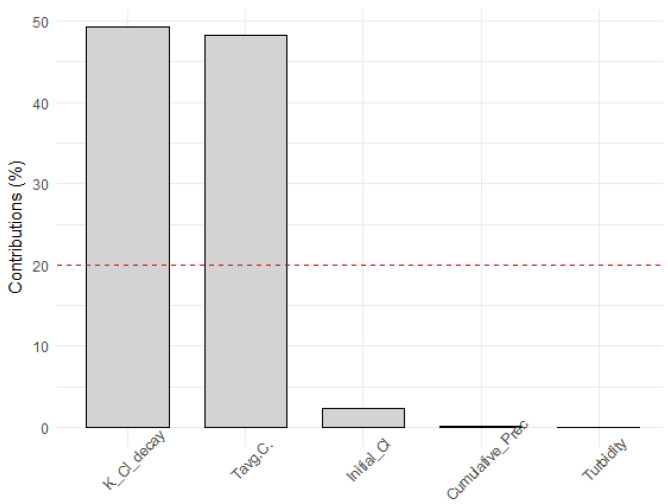
Contribution of variables to dimension 5. Variables with contributions below the dotted line are considered not significant for that dimension.

In this case, dimension one was related to the temperature, and dimension two was related to the initial chlorine concentration. Therefore, the decay constant was closely related to the temperature. Figure 10 shows the variables that had the most influence on dimension five, which were again the temperature and the chlorine decay, demonstrating the clear strong relation of the temperature on the decay constant.

Thus, it was concluded that the variable that had a higher effect on the decay constant was temperature. It was observed that the variables turbidity, precipitation, and initial chlorine did not excessively improve the fit between the decay constants of the model and the decay constants obtained. Therefore, only the temperature was used, since the effort required to obtain values for the rest of the parameters did not compensate the improvement of the model adjustment.

Experimental data from 2017 and *K_b_* obtained with the on-line calibration were used to calibrate the parameters of the two equations, an Arrhenius model (8) and a power model (9), to predict the temperature effect on the decay constant. The following Equations (10) and (11) show the results obtained.

(10) $$K_{b}=5.477\cdot 10^{-8}\cdot T^{1.524}\;(s^{-1})\;$$

(11) $$K_{b}=3950\cdot exp\left(-49873/RT\right)\;(s^{-1})$$

The fit obtained using the Arrhenius model and the power model were similar, although the Arrhenius one was slightly better. The Arrhenius model also determines the activation energy (J/mol), which is the minimum energy that the system needs for the reaction to take place. The ratio Ea/R obtained in this study was 5999 K, which is in accordance with other authors. For example, Courtis et al. [22] estimated 5388 K and 6701 K for two different water distribution systems, Powell et al. [23] obtained a range between 7500 and 9600 K, and Hua et al. [12] obtained a range between 8203 and 8727 K, depending on the type of water. The variability of the values for this ratio suggests that this is a water-specific parameter that might depend significantly on the natural organic matter composition [24].

**Figure 11:**
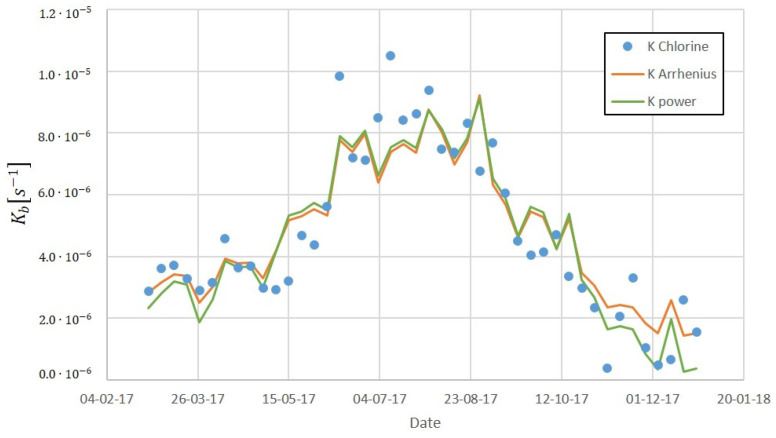
Chlorine decay constant fitting to temperature dependent models using data from 2017.

**Figure 12:**
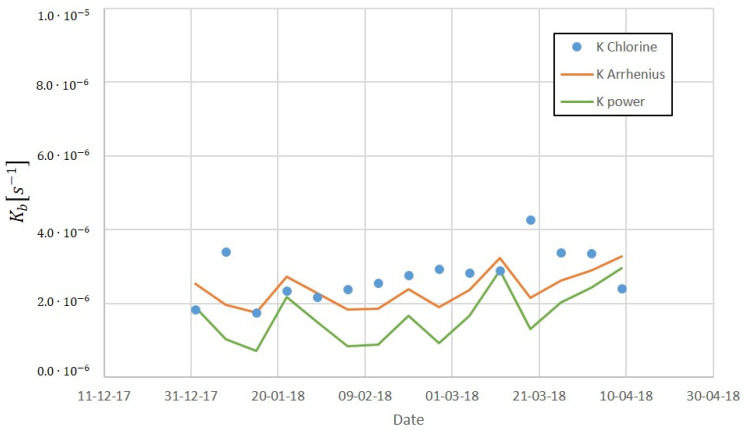
Chlorine decay constant forecast for the first days of 2018.

Figure 11 shows the fit of the two models to data from 2017, and Figure 12 their forecast for the first days of 2018. In these figures, K Chlorine is the chlorine decay constant determined previously, K power the constant determined following the potential model, and K Arrhenius the constant obtained from the Arrhenius model. As it can be seen, the Arrhenius model provides good predictions while using only the temperature as an input parameter.

## 4. Conclusions

The literature review shows that only the simplest models of chlorine decay are applied to water distribution networks where the hydraulic behavior is complex enough. Even so, these models are seldom used due to the lack of proper calibration. In this paper, the performance of a decay model was evaluated when the parameters were calibrated using state-of-the-art techniques.

The calibration was first carried out in the transport network, where the on-line data allowed an on-line calibration. The relation between the decay constant in the transport and distribution networks used analytical data and, thus, it could not be done on-line.

The prediction error in the validation data was 17% and quite similar to the error obtained for the training set (16%), which meant that there was no overfitting. The decay constant obtained changed during the year following the assumed dependence of the chlorine decay on the temperature. This result suggested the possibility of using available data for predicting this decay constant.

The principal component analysis determined that the temperature was the parameter with higher effect on the decay of chlorine. The chlorine decay constant was obtained using temperature as an independent variable. The obtained constants were compared with the data-driven model obtained in the on-line calibration, showing a high correlation. While the dominant dependence on the temperature is not a novelty, it is important to ensure this unique dependency, as it guarantees that other characteristics of the water source will not be relevant. This has been studied throughout one year, and the results obtained by both models are coherent.

This procedure could also be applied to other quality parameters, such as disinfection byproduct concentrations, which are currently under investigation by the authors. The variables analyzed for chlorine decay estimation are being studied for the trihalomethanes formation prediction.

The final aim of this study is to increase knowledge within the network in order to enable decision-making processes regarding chlorine dosing (quantity and frequency) both in the disinfection process and the boosting stations, in addition to identifying whether other control systems are required to ensure the continuous good quality of supplied water to the final user at a minimum cost.

## Data Availability

The data presented in this study are available on request from the corresponding author. The data is not publicly available because the owners want to keep track of its use, as it is sensitive information from a full-scale system (hydraulic models). Nevertheless, software for data treatment and chlorine decay model estimation and validation including nonsensitive data (model_estimation.m and model_validation.m) are available at https://cs2ac.upc.edu/en (18 July 2022) in Matlab format.

## Supplementary Materials

The following supporting information can be downloaded at: https://www.mdpi.com/article/10.3390/s22155578/s1, Table S1: Pseudocode to estimate the decay chlorine constant; Table S2: Pseudocode to estimate the chlorine decay constant in the distribution network.
