# Supplementary material for: Chlorine Concentration Modelling and Supervision in Water Distribution Systems
Source: Sensors (Basel). 2022 Jul 26;22(15):5578. doi: 10.3390/s22155578 (PMC9331035; doi:10.3390/s22155578)
Supplement: Supplementary file 1 [file sensors-22-05578-s001.zip › sensors-1741217-SI.pdf]

sensors

Supplementary Materials

Chlorine Concentration Modelling and Supervision in Water Distribution Systems

Ramon Pérez [1,2], Albert Martínez-Torrents [2], Manuel Martínez [2,3], Sergi Grau [4,5], Laura Vinardell [2], Ricard Tomàs [4], Xavier Martínez-Lladó [2] and Irene Jubany [2,*]

[1] Research Center for Supervision, Safety and Automatic Control, Universitat Politècnica de Catalunya, Rambla Sant Nebridi 10, 08222 Terrassa, Spain; ramon.perez@upc.edu
[2] Sustainability Area, Eurecat, Centre Tecnològic de Catalunya, Plaça de la Ciència, 2, 08243 Manresa, Spain; albert.martinez@eurecat.org (A.M.-T.); manuel.martinez@eurecat.org (M.M.); laura.vinardell@eurecat.org (L.V.); xavier.martinez@eurecat.org (X.M.-L.)
[3] Department of Mechanical Engineering, Universitat Rovira i Virgili, Av. Països Catalans 26, 43007 Tarragona, Spain
[4] Aigües de Manresa S.A., Plana de l'Om 6, 3r-3a, 08240 Manresa, Spain; sgrau@aiguesmanresa.cat (S.G.); rtomas@aiguesmanresa.cat (R.T.)
[5] Department of Mining, Industrial and ICT Engineering, Universitat Politècnica de Catalunya, Av. de les Bases de Manresa, 61-73, 08242 Manresa, Barcelona
* Correspondence: irene.jubany@eurecat.org; Tel.: +34-93-877-73-73

1. Calculation of added chlorine concentration

The concentration of the chlorine added in the network refereed to free chlorine (Cl_2) was calculated from the concentran of the reagent used, which was NaClO at 15 % as shown in the following equation:

$$Cl_{reagent} = \frac{15gNaClO}{100g\,water}\frac{1\,g\,water}{1\,ml\,water}\frac{1000ml}{1l}\frac{1molNaCLO}{74.45gNaClO}\frac{1molCl_2}{1molNaClO}\frac{70.9gCl_2}{1molCl_2} = 143gCl_2/l \qquad (1)$$

This equation is based on the following reaction:

$$Cl2(g) + H2O \rightarrow HOCl + HCl \qquad (2)$$

2. Algorithm for on-line decay constant calibration

The algorithm for the on-line calibration of the chlorine decay constant described in section 2.2 (article) are provided in pseudocode in Table 1. Varibles are consistent with those in section 2.2 (article).

Table S1. Pseudocode to estimate the decay chlorine constant.

Weekly Decay constant estimation	
Require:<br>$V_1, V_2, \Delta V_{Cl}, Q_1, Q_2, L, \emptyset, Cl_{reagent}$	Return: K_b
1: For $i = 1 : N_{weeks}$ do:	
2: *Evaluate* $\overline{V_1} = mean(V_1(week = i))$	
3: *Evaluate* $V_{pipe} = L \cdot \pi \cdot \left(\frac{\emptyset}{2}\right)^2$	
4: *Evaluate* $\overline{Q_1} = mean(Q_1(week = i))$	
5: *Evaluate* $\overline{RT_1} = \frac{\overline{V_1}+V_{pipe}}{\overline{Q_1}}$	
6: *Evaluate* $\overline{V_2} = mean(V_2(week = i))$	
7: *Evaluate* $\overline{Q_2} = mean(Q_2(week = i))$	
8: *Evaluate* $\overline{Q_2} = mean(Q_2(week = i))$	
9: *Evaluate* $\overline{RT_2} = \frac{\overline{V_2}}{\overline{Q_2}}$	
10: *Evaluate* $\overline{Cl_{added}} = \frac{\Delta V_{Cl}(week=i) \cdot Cl_{reagent}}{\int Q_2(week=i)}$	
11: *Evaluate* $\overline{Cl_1} = mean(Cl_1(week = i))$	
12: *Evaluate* $\overline{Cl_2} = mean(Cl_2(week = i))$	
13: *Evaluate* $K_b(i) \mid \overline{Cl_2} = \overline{Cl_1} e^{-K_b(\overline{RT_1}+\overline{RT_2})} + \overline{Cl_{added}} e^{-K_b(\overline{RT_2})}$	
12: End for	
14: Return: K_b	

3. Algorithm for decay constant estimation in the distribution network

The algorithm for the calibration process of the chlorine decay constant in the distribution network described in section 2.3 (article) are provided in pseudocode in Table 2.

Table S2. Pseudocode to estimate the chlorine decay constant in the distribution network.

Decay constant estimation for distribution network	
Require: K_b, Cl_{Sample}, *WDN Model*, ε *(finalisation criterion)*	Return: μ
1: *Initialise* μ<br>2: While: $\Delta MSE > \varepsilon$<br>3: *Simulate WDN Model with* $K_w^{dist} = K_b^{dist} = \mu \cdot K_b$<br>4: *Evaluate* $Cl_{Simulation}$<br>5: *Evaluate* $MSE = \frac{1}{N} \sum_N \left(Cl_{Sample} - Cl_{Simulation} \right)^2$<br>6: $\mu = \mu + \Delta\mu$<br>7: End while<br>8: Return: μ	
